# Epidemiological accountability: philanthropists, global health and the audit of saving lives

**DOI:** 10.1080/03085147.2018.1433359

**Published:** 2018-03-09

**Authors:** David Reubi

**Affiliations:** Department of Global Health & Social Medicine, King’s College London, Room 2.4, East Wing Building, Strand, London WC2R 2LS, United Kingdom.

**Keywords:** philanthropy, global health, audit, accountability, epidemiology, metrics

## Abstract

There have been concerns about the recent private turn and re-emergence of philanthropies in world health, with many worrying about philanthropies’ perceived lack of transparency and accountability. In contrast, I argue that while the private turn might have led to a decline in democratic or public accountability, it did not bring an end to all forms of accountability. Specifically, I suggest that philanthropists’ involvement in global health has led to the spread of another, new form of accountability: epidemiological accountability. The latter is a combination of two regimes of expertise and practices hitherto kept separate: audit and epidemiology. To substantiate this argument, I draw on my research on the Bloomberg Initiative – a global effort to reduce tobacco use spearheaded by the Bloomberg and Gates foundations.

In God we trust; everyone else bring data. (Michael R. Bloomberg, entrepreneur, public servant, philanthropist)

## Introduction

The shift from international to global health at the turn of the twenty-first century has been characterized by a private turn and the re-emergence of philanthropic foundations as major actors in world health, a trend symbolized by the Gates Foundation (Brown *et al*., 2006; Reubi, 2016a; Rushton & Williams, 2011). Many commentators have welcomed the arrival of these new actors, arguing that they bring with them much needed additional funding, a dynamic entrepreneurial spirit and innovative approaches from the business world (e.g. Bishop & Green, 2008; Moran & Stevenson, 2013). Others have been less sympathetic, levelling a range of critiques against the newcomers (e.g. Birn, 2014; McCoy, Kembhavi *et al*., 2009; McGoey, 2015). One particularly important concern for these critiques has been private foundations’ perceived lack of transparency and accountability. They point out how decisions about what should be funded and why are made by a small, restricted group of people behind closed doors. This, they argue, is problematic given the growing power of foundations in global health. It also begs the question of what interests are being pursued by this small group of people, many of whom come from the pharmaceutical industry. These critiques also stress how private foundations not only make little effort to engage in dialogue with experts and the public but actually actively discourage debate by promoting uncritical groupthink among global health specialists and officials.

This paper takes a different line. It argues that while the re-emergence of foundations in world health might well have led to a decline in what Rushton and his colleagues (2011, pp. 18, 157) call ‘democratic’ or ‘public accountability’, it would be a mistake to think that it has brought an end to all forms of accountability.^1^ Specifically, the paper suggests that the growing involvement of philanthropists in global health has led to the emergence and spread of another, new form of accountability, which I term epidemiological accountability. This new form of accountability is the result of the coming together of two regimes of expertise and practices that were hitherto kept separate. The first is contemporary cultures of audit. A collection of reporting routines, performance indicators, accounting firms and management experts, these cultures were progressively articulated in the twentieth century to govern commercial life before being redeployed to administer public life under the banner of New Public Management (Power, 1997; Shore & Wright, 2015). The second regime is the forms of knowledge and techniques associated with modern epidemiology. These forms – from vital registration systems and health surveys to national statistics agencies and epidemiologists – were articulated from the nineteenth century onwards as part of the biopolitics of population that characterizes most modern societies (Desrosières, 1998; Foucault, 1998).

To develop and substantiate this argument, the paper draws on extensive, multi-sited archival and fieldwork on the Bloomberg Initiative – an international effort spearheaded by Bloomberg Philanthropies and the Gates Foundation to reduce tobacco use in the developing world.^2^ After giving a short account of the Bloomberg Initiative, the paper goes on to identify and analyse how the expertise and practices of audit and epidemiology shape and inform the design and administration of the Initiative. It also explores the ways in which the expertise and practices of audit and epidemiology combine in the overall assessments and public presentations of the Initiative carried out by both the Bloomberg and Gates foundations at regular intervals. Moreover, the paper also studies some of the pathways along which the values and methods developed by both Bill Gates and Michael Bloomberg in their businesses have spilled into their philanthropic work and led to the emergence of epidemiological accountability.

## The private turn in global health and the decline of democratic accountability

The last 20 years have seen a profound rupture in the organization and management of world health, marked by the shift from international to global health (Brown *et al*., 2006; Gaudillière, 2014; Reubi, 2016a). Starting in the 1990s, a growing number of international policy-makers, development bureaucrats and public health experts began increasingly to talk about ‘global health’ instead of ‘international health’ or ‘health development’. Drawing on the globalization theories that were then predominant, these commentators believed that the world was undergoing a process of economic, political and social integration. For them, trade liberalization and a revolution in communication and transportation technologies were leading to ever-growing flows of information, goods, capital and people across political and geographical boundaries. Inherent to these processes of globalization, they warned, were new types of threats to human health, from the rapid propagation of infections enabled by affordable air travel to the global spread of fast foods, alcohol and cigarettes made possible by the abolition of trade barriers. For these commentators, the existing international health architecture centred on the nation-state was not equipped to deal with these new problems. Indeed, their transnational nature meant that these new types of threats were immune to the efforts of individual nation-states and required new, global forms of thought and action. These included global legal norms like the WHO's Framework Convention on Tobacco Control, new international financing mechanisms like The Global Fund to Fight AIDS, Tuberculosis and Malaria and global epidemiological data like the Global Burden of Disease estimates.

The incapacity of the nation-state to deal with the new health threats associated with globalization was, in the eyes of these commentators, further compounded by the wider critique of the state as incompetent and wasteful articulated by neoliberal thinkers in the latter half of the twentieth century (Reubi, 2013, 2016b). This led them to promote not only new global forms of action, but also interventions by non-state (or private) actors, including non-governmental organizations, public–private partnerships and, importantly for us, philanthropies. Of course, as the work of the Rockefeller Foundation's International Health Division during the first half of the twentieth century reminds us, the involvement of philanthropies in the administration of world health is not new (Birn, 2014; Farley, 2004). However, the private turn that seems so characteristic of contemporary global health has certainly led to the re-emergence of philanthropic foundations as major actors in world health (Rushton & Williams, 2011; Youde, 2013). The creation of the Bill and Melinda Gates Foundation in the early 2000s is perhaps the most visible sign of this recent trend, but there are many other philanthropies that are active in global health today, from the Carso Health Institute established by Mexican billionaire Carlo Slim to the Clinton Foundation and Bloomberg Philanthropies. These philanthropic organizations have been highly influential in world health over the last 15 years, funding biomedical research, launching public health initiatives, reconfiguring overseas health development aid and brokering new alliances between international organizations, the pharmaceutical industry, governments and NGOs (McGoey, 2014; Moran & Stevenson, 2013; Youde, 2011).

Predictably, the literature is divided on the advantages and drawbacks associated with foundations’ growing influence in world health. Some commentators have celebrated the arrival of these new actors, claiming that they ‘have added dynamism, credibility and attractiveness to global health’ (*The Lancet*, 2009, p. 1577; e.g. Bishop & Green, 2008; Moran & Stevenson, 2013). When discussing foundations’ contributions to global health, they generally mention the increased sources of funding, a new entrepreneurial spirit concerned with efficiency, leaner, more flexible decision-making structures and innovative approaches imported from the business world. Other commentators have been more critical of philanthropies’ contributions to global health (e.g. Birn, 2014; McCoy, Kembhavi *et al*., 2009; McGoey, 2015). Some of these critiques believe that the work of foundations in global health is an attempt by business elites to ‘cover for the deleterious effects of global neoliberal capitalism’ (Youde, 2013, p. 152). Many also argue that, unfortunately, philanthropists tend to favour ‘biomedical and largely pharmaceutical-based responses to global health problems’ that ignore the underlying social determinants of diseases and pay little attention to the local context (Rushton & Williams, 2011, p. 10; see also Birn, 2014).

More significantly for the argument attempted here, these critiques have complained that foundations ‘lack transparency and accountability’ (McCoy & McGoey, 2011, p. 152), with some even claiming that philanthropies are ‘among the most unaccountable organisations in democratic societies’ (Hesselmann, 2011, p. 236). To start with, they point out how, in most foundations, decisions about what is a funding priority, who to partner with and why are ‘in the hands of very few’ people and made ‘behind closed doors, hidden from the public's eye’ (Stuckler & Siegel, 2011, pp. 136, 139). Given the growing power of foundations in the field of global health, the secret nature of this decision-making process and the restricted number of those influencing it have generated serious anxieties among critical commentators. So, for example, the editors of *The Lancet* have recently complained about the opacity and arbitrariness of the principles guiding the work of the Gates Foundation:

There is also a serious anxiety about the transparency of the Foundation's operation. What are the Foundation's future plans? It is hard to know for sure. The first guiding principle of the Foundation is that it is ‘driven by the interests and passions of the Gates family’. An annual letter from Bill Gates summarises those passions, referring to newspapers, articles, books and chance events that have shaped the Foundation’s strategy. Is such a whimsical governance principle good enough? (*The Lancet,* 2009, p. 1577)

Similarly, others have expressed concerns about whose interests are being served by the select few who make the decisions for private foundations. Thus, sociologist Linsey McGoey (2015) has queried whether the strong presence of former drug industry executives among the decision-makers at Gates has contributed to the foundation's bias for pharmaceutical solutions, while political economist David Stuckler and his collaborators have suggested there could be a link between Gates’s refusal to tackle non-communicable diseases and the foundation's substantial investment in Coca-Cola and other corporate giants of the agro-food sector (Stuckler & Siegel, 2011).^3^ Furthermore, those critical of philanthropies’ lack of transparency and accountability also point out that private foundations make little effort to engage in dialogue with and respond to constructive criticism from experts in the field, grant recipients and the general public. As political scientist Jeremy Youde explains in his study of the Clinton Foundation:

[Private philanthropies] have disproportionate influence, yet the public has next to no opportunity to express its opinions or voice concerns about their programmes. They replace a mass democratic voice with a top-down autocratic one … [There are no] opportunities for both donors and recipients to voice their concerns and … engage in public dialogue about priorities and appropriate approaches … Private philanthropies like the Clinton Foundation … assume the voice of those they claim to represent instead of allowing them their own voice. (Youde, 2011, p. 176)

Moreover, these critiques claim that, not content with failing to generate avenues for dialogue, many foundations are actively ‘discouraging debate’ by ‘creating a cartel mentality’ and ‘promoting groupthink’ among global health expert and officials (Youde, 2013, p. 151). For example, global health expert and physician David McCoy has described how, by aggressively pushing its own agenda, the Gates Foundation has led to ‘self-censorship and the stifling of diverse views among scientists’ who are now afraid to voice any sort of criticism for fear of being denied any funding (McCoy & McGoey, 2011, p. 153; see also Birn, 2014; Stuckler & Siegel, 2011). For these critiques, this lack of accountability and transparency on the part of private foundations in the field of global health ‘represents a significant challenge to democracy and principles of good governance’ (McCoy & McGoey, 2011, p. 157). To address this issue, they have repeatedly called for the adoption of better mechanisms of public accountability and standards of transparency. So, for example, the editors of *The Lancet* implored the Gates Foundation to:

Improve your governance. Visibly involve diverse leaders with experience in global health in your strategic and operational stewardship … Be more transparent and accountable in your decision making … Listen and be prepared to engage with your friends. (*The Lancet*, 2009, p. 1577)

## Epidemiological accountability

While the private turn and re-emergence of foundations in world health might well have led to a decline in this type of ‘democratic’ or ‘public accountability’ (Rushton & Williams, 2011, pp. 18, 157), it would, however, be a mistake to think that it has brought an end to all forms of accountability. Indeed, as I suggest here, the growing involvement of philanthropists like Bloomberg and Gates in global health has led to the emergence and spread of another, new form of accountability, which I term epidemiological accountability. As the paper describes in more detail below in relation to the Bloomberg Initiative, this new form of accountability brings the expertise and practices of audit together with those of modern epidemiology.

As Michael Power (1997) and others (e.g. Merry, 2011; Shore & Wright, 2015; Strathern, 2000) have argued, the expertise and practices of audit – reporting and verification procedures; performance indicators; accounting experts; management firms – have become central to the government of life. Progressively articulated during the twentieth century as part of attempts to protect investors against fraud and efforts to improve productivity and product quality, these forms of expertise and practices were initially primarily concerned with the management of commercial and corporate life. It is only from the 1980s onwards that, spurred by New Public Management theories, they spilled into and re-shaped the organization and administration of public life, from education and health care to scientific research and international development. New Public Management theories have also been critical in re-inscribing these forms of expertise and practices within a complex web of political values and beliefs. These include demands for fiscal restraint, misgivings about the state's incompetence and a belief in market efficiency, as well as appeals for increased accountability and value-for-money.

The expertise and practices of modern epidemiology have a long genealogy (Reubi, 2017). An important, early development was the birth of vital statistics in the nineteenth century (Desrosières, 1998; Szreter, 1991). This quantitative knowledge about a population's births, marriages and deaths was made possible by a combination of national statistics bureaucracies, public health reformers and new quantification practices like vital registrations systems and censuses. More generally, it was also made possible by a reconfiguration of political power, from a medieval sovereign concerned with God's natural order to a modern government dedicated to improving the condition of the population (Foucault, 2009). Indeed, this new form of power involved a new biopolitics of population devoted to fostering biological life and guided by scientific and, especially, statistical truth (Foucault, 1998; Porter, 1995). This assemblage of vital statistics and biopolitics was partly reconfigured by the rise, in the twentieth century, of surveillance medicine articulated around the community dispensary, unhealthy lifestyles and a politics of social solidarity (Armstrong, 1983, 1995). Specifically, surveillance medicine spurred a shift from the old public hygiene concerned with the environment and infections to the new public health focused on individual behaviour and chronic diseases. It also led to the development of a new way to conceive and quantify the life of the population – the social survey, which measured the biological, social, economic and political dimensions of unhealthy lifestyles. A product of the new academic discipline of epidemiology taught at public health schools after the 1930s, this new quantification practice was built around sophisticated statistical techniques and qualitative sociological methods.

## The Bloomberg Initiative

To illustrate and further elaborate this new, hybrid form of accountability marrying the expertise and practices of audit and modern epidemiology, I draw on my research on the Bloomberg Initiative. Whether this epidemiological accountability informs other, similar philanthropic global health efforts is something that only further research can tell. But, given that the Bloomberg Initiative and other, similar philanthropic efforts share many of the same forms of knowledge, institutions and techniques, it is reasonable to expect that a similar form of accountability also shapes these other efforts (e.g. Adams, 2016; Mahajan, in press; McCoy, Jensen *et al*., 2013). At the very least, one can assume that these other efforts are informed by forms of accountability that, to draw from Wittgenstein (2009, p. 36), have strong ‘family resemblances’ to the epidemiological accountability at work in the Bloomberg Initiative.

**Figure 1:**
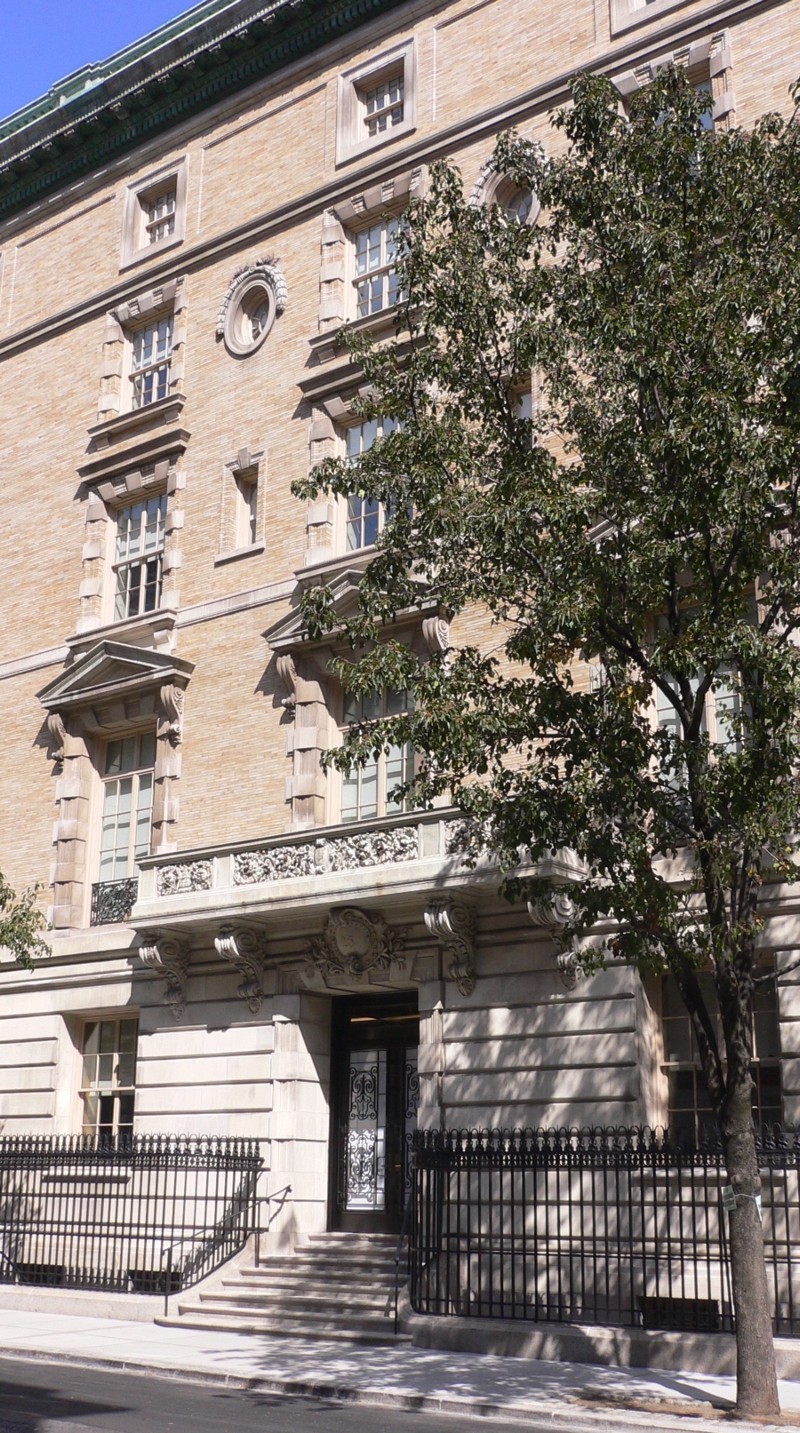
Headquarters of Bloomberg Philanthropies located off Park Avenue in New York City's Upper East Side.

The Bloomberg Initiative was launched in 2007 by businessman and then mayor of New York City Michael Bloomberg and his charity Bloomberg Philanthropies with the aim to reduce tobacco use in the developing world (Bloomberg Philanthropies, 2011; see Figure 1). A year later, the Bill and Melinda Gates Foundation became an official partner of the Initiative, adding an extra US$125 million to the almost US$1 billion invested by Bloomberg (Bloomberg Philanthropies, 2016; Gates Foundation, 2015a). For Bloomberg and the Gateses, the Initiative is part of their wider commitment to ‘give back’ to the community and help ‘make a better world’ (Bloomberg, 2001, p. 242; Gates & Gates, 2010, p. 2). The Initiative builds on the anti-smoking campaign that the then New York Health Commissioner Tom Frieden and his team ran in the city during Bloomberg's mayoralty. For Bloomberg and Frieden, who had been tasked by the Mayor with designing the Initiative, the latter was a great opportunity to showcase their work in New York at a time when, following the adoption of the WHO Framework Convention on Tobacco Control, there was growing momentum in the international fight against smoking (Reubi & Berridge, 2016). In keeping with Frieden's original blueprint, the Initiative comprises three hierarchically ordered groups of organizations. At the apex are the Bloomberg and Gates foundations, whose experts decide the overall strategy, objectives and funding allocation. It is, for example, these experts who determined that the initiative should focus on a well-defined package – the so-called MPOWER package – of tobacco control policies that includes: advertising bans; smoke-free environments; cessation programmes; tobacco taxation; public health campaigns; and warning labels (WHO, 2013a, 2013b). At the bottom are local advocacy groups and government agencies whose role is to ensure that these policies are put in place and implemented. In the middle are a small number of privileged partners that act as a sort of relay between the two philanthropies and the local organizations. These privileged partners include the WHO's Tobacco Free Initiative, the US Centres for Disease Control (CDC), the Johns Hopkins School of Public Health, and some, mostly American charities like the Campaign for Tobacco-Free Kids, the International Union against Tuberculosis and Lung Disease and, to a lesser extent, the American Cancer Society (Bloomberg Philanthropies, 2011; Gates Foundation, 2009). The role of these organizations, whose tobacco control work is directly funded by Bloomberg and Gates, is two-fold: set up a global monitoring system to track the evolution of tobacco use and tobacco control policies; and support the work of local organizations through technical assistance, capacity building and small grants programmes.

### Expertise and practices of audit

As with some other philanthropic efforts (e.g. Adams, 2016; Mahajan, in press; Youde, 2013), the expertise and practices of audit powerfully inform the organization and management of the Bloomberg Initiative. Grantees working for the Initiative repeatedly noted this fact in our interviews:

The Americans [Bloomberg and Gates] love accountability, a lot of it. Accountability and transparency the whole nine yards. So, you need to check that your story is actually straight.

The Bloomberg people are just here all the time, reviewing and checking our work all the time.

Reporting routines are an important aspect of the audit culture at work within the Bloomberg Initiative. The organizations that receive funds from the Initiative, either directly from the Bloomberg and Gates foundations or indirectly via the small grants schemes managed by the privileged partners, are required to report on a regular basis. While exact reporting requirements can vary from one scheme to another, the general pattern is the same throughout the Initiative. For each project, grantees will have to deliver monthly written activity reports, quarterly intermediary written reports and one final written report. In addition, grantors will phone grantees up to twice a week to ask for oral updates on the project. As a seasoned grant administrator in the field explained to me, this amount of reporting is exceptional:

Bloomberg is a unique donor. Most traditional donors, I mean development agencies like USAID, they kind of write a cheque and say: ‘tell us in a year what happens’. Bloomberg are sitting in our laps all the time. They are right in everybody's face always. We report quarterly on our budget which is not the case for any other donor. We have monthly reports back to them on all of our capacity building and technical assistance that we’re doing. We have annual reports to do. I’m on the phone with them ten times a month. It is a very unusual relationship for us. It has been a bit of a culture shock.

**Figure 2:**
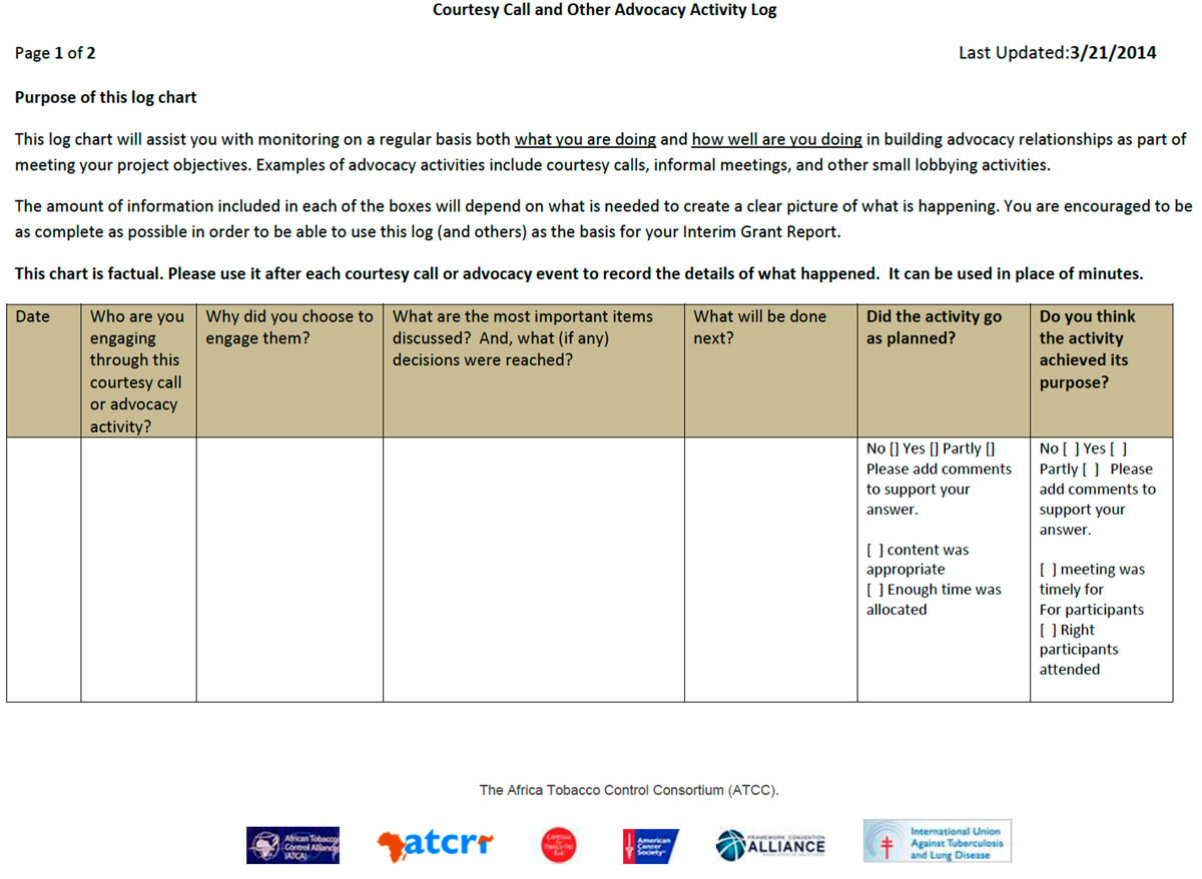
Advocacy Activity Log developed for the African Tobacco Control Consortium led by the American Cancer Society and funded by the Gates Foundation. Permission to reproduce granted by the African Tobacco Control Alliance.

The reports will generally comprise two types of information. First, they will contain financial information, including a budget indicating the money spent to date together with the original receipts and invoices. Second, the reports will also contain information on all the grantees’ activities that are relevant to the project: the studies commissioned and the experts consulted; the total of information leaflets printed and distributed; the number of workshops organized and the people trained; the lobbying activities from phone calls to journalists to courtesy visits to politicians; and so on. To ensure that this information is collected, grantees are required to record any relevant activity on *ad hoc* log sheets, which generally contain a series of boxes to mark the date, describe the activity, indicate the people involved, explain how successful it was and discuss what will be done next (see Figure 2). As one grantee explained:

We have these reporting logs. There is a log for industry monitoring, one for project progress, one for courtesy calls, one for the status of the law, oh there are so many logs … For accountability and for follow up you keep filling in these logs. So, I go to see this MP, he said X and Z, you fill it in. Or, there was this tobacco industry interference that was reported in the media today, this is how we would want to counter it. Or you did a workshop, you write in the log how many people you called, how many emails you sent, how many persons attended. It's quite a bit of work … But it helps you remember things and makes writing the reports much easier.

The reports allow grantors to monitor the project's progress and check whether the key milestones identified in the grant agreement are being achieved. In the world of the Bloomberg Initiative, it is only if these milestones are achieved that the next tranche of funding can be disbursed. The reports also allow grantors to identify a problem early on and attempt to rectify it, be it by providing technical assistance or extra training, allowing the reallocation of an unspent budgetary post to another task or agreeing to a no-cost extension. It is only if nothing can be done to rectify the problem that grantors will consider terminating a project, something that happens surprisingly rarely.

Some of these same functions are also fulfilled by another important element of the Bloomberg audit culture: the inspection. At regular intervals, grantors will send out independent auditors to carry out on-site visits to inspect grantees and evaluate their projects. A key purpose of these visits is to meet grantees, check their organizations’ financial management and operational systems and teach them about the funder's procedures. As one auditor explained to me:

A site visit is … the chance to meet the grantees. Just walk around the office and familiarise ourselves. Know that we are working with a *bona fide* partner … It is about assessing the organisation's finance management system, to ensure it is effective, efficient. We audit their reports, the time sheets, rental agreements, accounting system. We check receipts, the work plan, the budget, that everything is in order … We check how they manage the office, how they file and send documentation, etc. It is also a capacity building moment, to tell them about our procedures.

Another aim of these audits is to verify the activities reported by the grantees, as one grantee explained to me:

They sent someone to evaluate my report. And they said: ‘Oh you said you worked with these people, can we talk to them?’ … So, I took them around to about thirteen major partners – ministry of health, journalists, civil society, members of parliament – who I said I had been dealing with in my reports. Then they talked to them and asked: ‘Is it true?’

The audit culture that informs the Bloomberg Initiative also brings with it accountants and management specialists – novel forms of labour and expertise in the field of international tobacco control that previously was dominated by physicians, public health specialists, lawyers, economists and anti-smoking activists (Reubi & Berridge, 2016). To start with, grantors generally use management experts and accountants to check the reports sent by the grantees and carry out on-site inspections. So, for example, the American Cancer Society hires an external consultant, a small accountancy and project management firm based in Nairobi, to audit its grantees working on tobacco control in Africa. Likewise, most project management and financial compliance officers working for either the American Cancer Society or the Campaign for Tobacco-Free Kids have a formal training in business studies or development management. Moreover, grantors strongly encourage grantees to hire professional accountants and to themselves acquire basic project management skills by taking some relevant courses. As one grantor pointed out:

Our grantees are great [tobacco control] advocates but they often have no idea how to do any kind of business or accounting or office management. When we note this during an inspection, we recommend that they hire an accountant or an office manager. Or we encourage them to undergo some kind of training in project management. And we provide them with the necessary extra funding to do this. As a result, they’ve really grown in terms of their capacity for managing money and managing projects. In terms of being a real organisation.

### Expertise and practices of epidemiology

The design and management of the Bloomberg Initiative is permeated not only by the expertise and practices of audit but also by those of modern epidemiology. A clear sign of the centrality of epidemiological expertise and practices for the Initiative is the critical part played by professional epidemiologists in devising and administrating it. Tom Frieden and Kelly Henning, two medical doctors trained in epidemiology at the CDC, are exemplary in that respect: as mentioned earlier, Frieden, now Director of the CDC, was in charge of setting up the Initiative when he was Health Commissioner of New York City; and Henning, as Director of International Health Programmes at Bloomberg Philanthropies, helped Frieden design the Initiative and now directs it (Frieden & Bloomberg, 2007). Likewise, Kathy Cahill, a public health specialist who worked as Director for Planning, Policy and Evaluation at the CDC, was responsible for devising the Gates Foundation's tobacco control strategy and partnership with the Bloomberg Initiative.

It is these and other epidemiologists who, applying their knowledge and methods, translated Bloomberg and Gates’s desire to save lives into a project with a clear focus, strategy and targets (Bloomberg Philanthropies, 2011; Gates Foundation, 2009). To start with, they suggested focusing the Initiative on tobacco use, pointing out that recent epidemiological studies like Christopher Murray's work on the Global Burden of Disease and Richard Peto's analyses on global smoking-attributable mortality showed that tobacco was now the leading cause of death worldwide (e.g. Lopez *et al*., 2006; Mackay & Eriksen, 2002; Peto & Lopez, 2000). As two epidemiologists involved in the design of the Initiative recalled:

The decision to focus on tobacco came entirely from the data … We were looking for an area related to a very large number of deaths worldwide … So, we looked at the numbers and analysed them. They showed that smoking was now the world's leading single cause of death with over six million deaths per year compared with three for AIDS, two for tuberculosis, one for malaria … We knew our focus had to be tobacco.

Bill Gates was convinced by the numbers. He has a very mathematical mind. He just read all the information. He read the Global Burden of Disease. He read the Tobacco Atlas … And he did the maths. He saw the number of lives that can be saved. That is why he became interested in tobacco and teamed up with Bloomberg.

By making clear that the smoking epidemic was concentrated and rapidly expanding in the developing world, the same studies also convinced epidemiologists working for Bloomberg and Gates that the Initiative should centre on low- and middle-income countries (Reubi, 2016a). Similarly, they made it possible for these epidemiologists to compute the Initiative's overall target of reducing the global smoking prevalence rate below 20 per cent and ‘preventing 100 million deaths’ by the year 2020 (Frieden & Bloomberg, 2007). In the same way, Tom Frieden and his colleagues decided to make the MPOWER policies the focus of the Bloomberg Initiative because they had been proved to be ‘effective’ by public health research (Frieden & Bloomberg, 2007; Gates Foundation, 2009).

**Figure 3:**
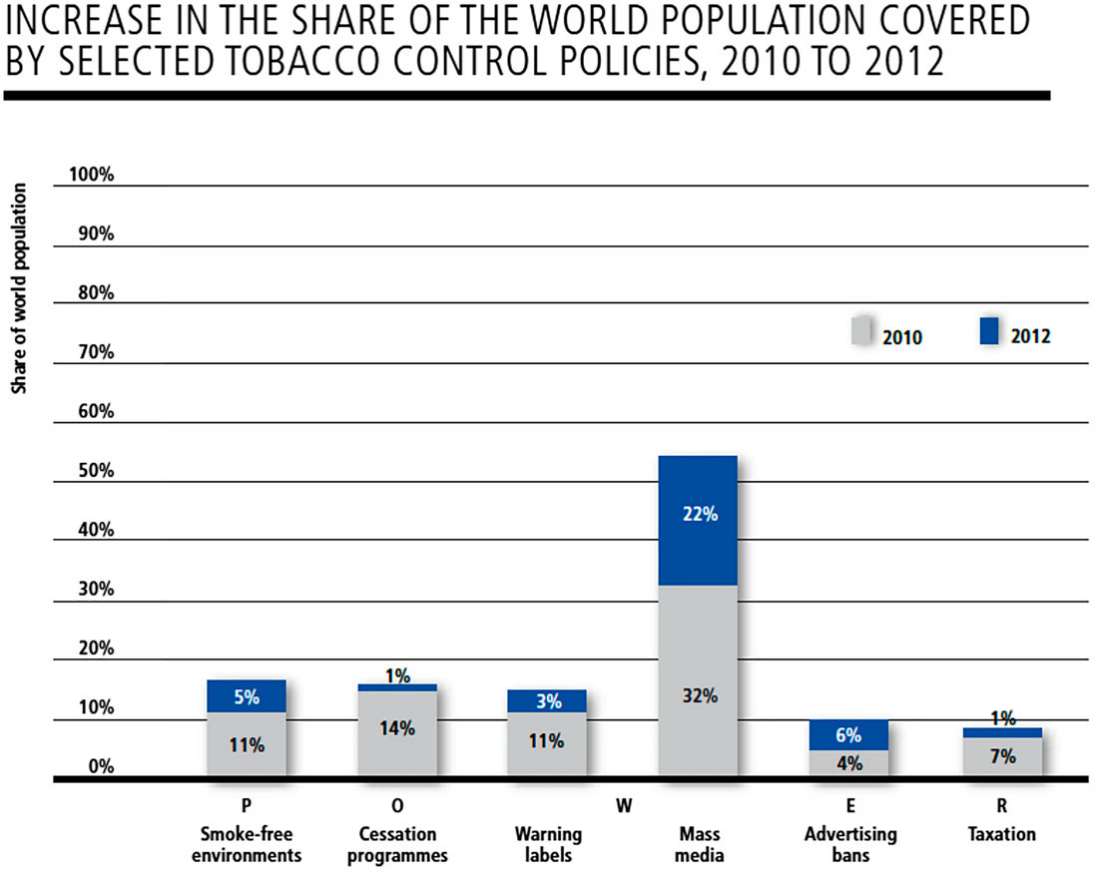
Graph taken from the WHO's 2013 Report on the Global Tobacco Epidemic showing the percentage of the world's population covered by MPOWER policies. Permission to reproduce granted by the WHO.

The epidemiologists who helped devise the Bloomberg Initiative also played a key role in setting up a global surveillance and monitoring system to follow the Initiative's progress. As one of these epidemiologists clarified for me, the system is built around ‘two major measurement strategies’. The first is the Report on the Global Tobacco Epidemic (GTER). Compiled by the WHO every two years, the report monitors the worldwide progress of tobacco control policies by counting the number of countries that have implemented and the percentage of the globe's population covered by at least one of the MPOWER policies (WHO, 2009, 2011, 2013a; see Figure 3). The second is the Global Adult Tobacco Survey (GATS). Run by the CDC in collaboration with national statistic agencies, the GATS is a household survey that monitors adult tobacco use and tracks key tobacco control indicators such as smoke-free environments and advertising bans (CDC, 2009). At the time of writing, the GATS, in either its full or abridged version, had been completed once in about 70 countries and repeated a second time in two countries (CDC, 2015). The longer-term aim, of course, was to have an ever-larger number of countries carrying out the GATS at regular intervals to make it possible to trace the fluctuation in tobacco use across the globe and over time. For Frieden and his colleagues, this two-fold surveillance and monitoring system was deemed critical for the effective management of the Initiative. They believed that such a system was critical to determine whether the strategies used were working and whether the targets identified were being reached. As they explained in an information leaflet:

Surveillance and monitoring are important public health … tools. They provide critical information to strengthen programmes and policies, and to evaluate their effectiveness. ‘If you can't measure it, you can't manage it.’ (CDC, 2011, p. 1)

### Combining audit and epidemiology

It is standard policy for both the Bloomberg and Gates foundations to continually evaluate the projects in which they invest. When describing their ‘approach’ to giving in their publications, Bloomberg Philanthropies (2015, p. 7) always stress the value they attach to ‘continually measur[ing] progress’. Likewise, the Gates Foundation (2015b, p. 1) emphasizes in its policy documents that ‘achieving our ambitious goals requires rigorous evaluation so we and our partners can continually improve’. It is this shared evaluation policy that has led the two philanthropies to regularly assess the Bloomberg Initiative and its overall impact. As one of the experts working for the Initiative explained to me in an interview, these regular assessments are ‘about accountability and good governance’. More specifically, they enable the two foundations to determine progress and analyse what is working and what is not and needs to be adjusted in order to improve the Initiative's overall impact. As Bloomberg Philanthropies (2013, p. 3) explain, we need ‘to harness the power of data’ to ‘understand impact, evaluate results and improve performance’. In a similar spirit, the Gates Foundation explains that:

Evaluation [is] a collaborative learning tool that provides us and our partners with feedback so we can learn, adjust and decide how best to achieve outcomes … [It] provides feedback on what is and is not working. (Gates Foundation, 2015b, pp. 2–3)

In addition, the regular assessments carried out by the two foundations also provide them with the necessary information to showcase their work to the global health community and beyond in annual reports, public presentations and media briefings (e.g. Bloomberg Philanthropies, 2011, 2015).

**Figure 4:**
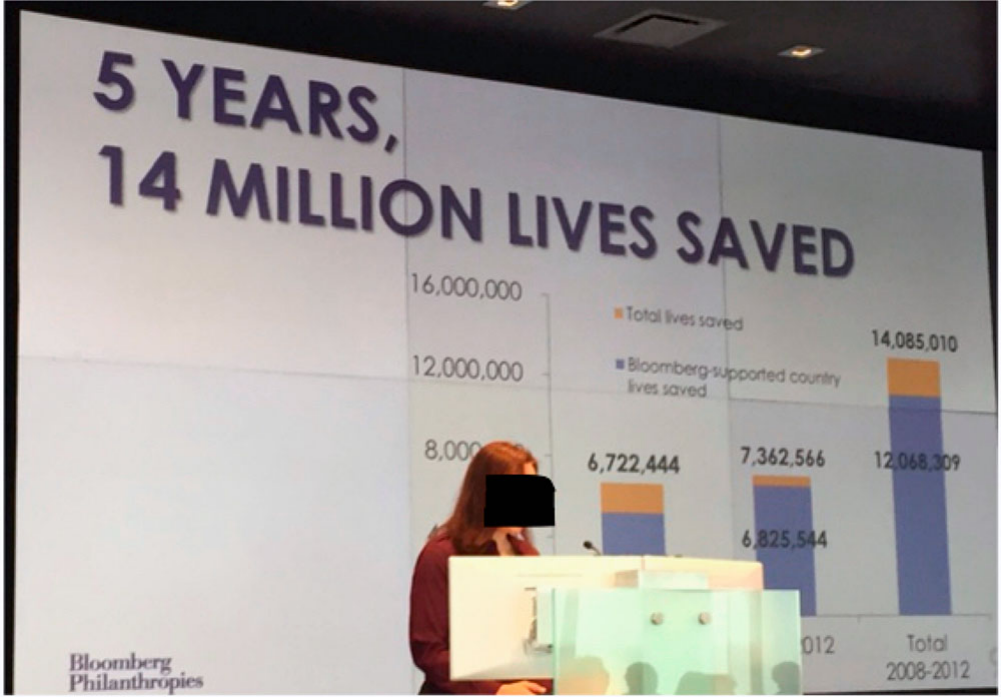
A Bloomberg Philanthropies senior health expert presenting the Initiative to grantees at the New York Headquarters of Bloomberg L.P.

Both the Bloomberg and the Gates foundations like to think of their evaluation protocols as quasi-scientific processes characterized by systematicity, objectivity and rigour. So, for example, in an interview with me, an expert at Bloomberg Philanthropies explained that the regular assessment of their projects was based on ‘very rigorous monitoring and surveillance’. Likewise, in a recent document on its evaluation policy, the Gates Foundation (2015b, p. 1) suggests that an evaluation is a ‘systematic, objective assessment of an ongoing or completed project or partnership’. The same document also indicates that an evaluation will draw on ‘many different types of evidence’, including: ‘partner monitoring data, grantee reports, modeling, population-level statistics and other secondary data’ (Gates Foundation, 2015b, p. 3). This is certainly characteristic of Bloomberg and Gates’s regular assessments of the Initiative, which are built around a combination of various forms of evidence associated with the expertise and practices of audit and epidemiology outlined earlier, including grantees’ reports, the GATS and the GTER. In official documents and presentations, these different forms of evidence are generally organized in three separate categories. The first category is ‘investments’ and generally corresponds to the total amount of money that Bloomberg and Gates have poured into the Initiative. So, for example, in one of its earlier report on the Initiative, Bloomberg Philanthropies (2011, pp. 1, 11) reminded readers that it had made ‘a $375 million investment’ to which Gates had added a ‘commitment of $125 million’. The second category is ‘actions’, which is the summation of all the different activities carried out and recounted by grantees in their reports. As a senior public health expert from Bloomberg Philanthropies elaborated during a presentation (see Figure 4) to a group of grantees:

When we are reporting back to Mike [Bloomberg] about our progress and our success in the Initiative … we talk about the training of journalists and how many stories have come out in support of tobacco control around the world; we look at all of the efforts you were involved in; and we count all of these on a regular basis.

**Figure 5:**
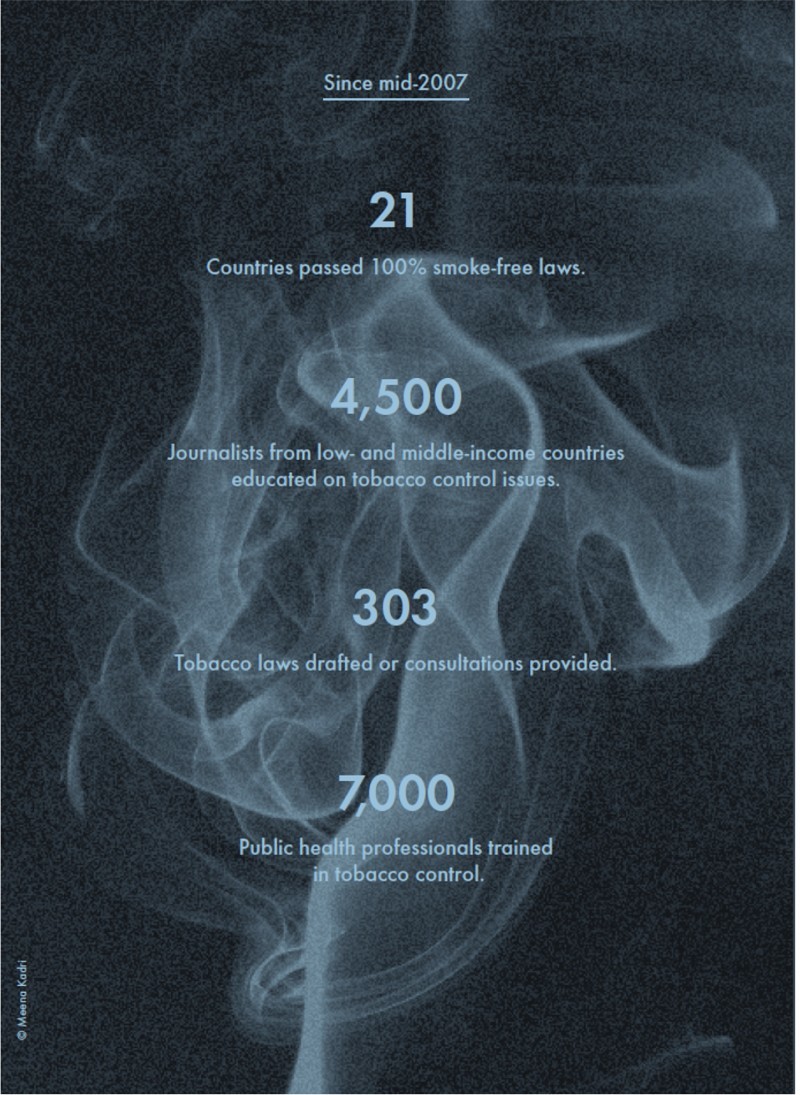
Image taken from a 2011 report by Bloomberg Philanthropies. Permission to reproduce granted by Bloomberg Philanthropies.

A good illustration of these counting practices can be found in a recent Bloomberg Philanthropies (see Figure 5) publication:

To date, 300,000 individuals … have completed the GATS … Funded partner organizations and in-country grantees [have]: … completed air quality monitoring studies in 34 countries; … developed a technical guide … [on] warning labels; launched 64 anti-tobacco … mass media campaigns in 18 countries, viewed by 643 million people; … conducted workshops with journalists from 28 countries on tobacco control issues; … provided legal expertise … [in] 55 countries; … [and] published 13 white papers on tobacco economics. In addition, … almost 300 senior-level government and civil society representatives from 34 countries have been trained through an annual intensive leadership program for tobacco control [and] more than 5,000 [individuals] from 172 countries participated in a free online training. (Bloomberg Philanthropies, 2011, pp. 14–25)

The third and last category is ‘outcomes’ or ‘impacts’. As an epidemiologist working for the Initiative explained to me, the ‘ultimate impact is reducing deaths’. In the absence of reliable data on global smoking-attributable mortality, the Initiative uses computer simulation models, such as the ‘SimSmoke’ model elaborated by American economist David Levy and his colleagues, to estimate the number of lives saved through its investments and activities (e.g. Dubray *et al*., 2015; Levy, Ellis *et al*., 2013). These models start with the numbers of activities reported by grantees and two measures of intermediate impacts – the number of countries that implemented one or more MPOWER policies, as measured by the WHO's GTER; and global smoking prevalence, as measured by the CDC's GATS. From there, they estimate the number of lives saved using a combination of assumptions about the effectiveness of single tobacco control policies, counterfactual ‘no-intervention’ or ‘status quo’ scenarios and baseline data on mortality rates and relative risks of death (Levy, Chaloupka *et al*., 2002; McCoy, Jensen *et al*., 2013). Usually, these models and the causal links between investments, activities and outcomes are taken for granted by experts working for the Initiative. So, for example, in the presentation to grantees mentioned earlier (see Figure 4), the senior expert from Bloomberg Philanthropies began by reiterating the amount of money invested by Bloomberg and Gates, then enumerated some of the activities carried out as part of the Initiative and, in conclusion, stated that:

We have made quite a bit of progress in the eight years since we started. We have had quite an impact. There has been a lot of success in recent years as the growing numbers of countries having adopted one MPOWER policy shows … And we estimated that the lives saved based on the policy progress we have made … We had expected some increases … but we were absolutely astounded … to see that all the policy progress made … had doubled our lives saved estimates, from 7 to 14 million.

While the aim of this paper is not to expose the shortcomings of the quantification techniques used in the Bloomberg Initiative, it is still worth noting here that the methodology and computer simulation models underpinning ‘saved lives’ metrics are not without problems. Indeed, as McCoy, Jensen *et al*. (2013, pp. 2, 5) have shown in relation to The Global Fund to Fight AIDS, Tuberculosis and Malaria, such metrics tend to be ‘overestimates’ because of the many ‘extrapolations, assumptions and generalisations’ on which they are based (see also Dubray *et al*., 2015; Levy, Ellis *et al*., 2013). To start with, McCoy and colleagues point out that the numbers that are entered into the models and serve as the base for computing ‘saved lives’ metrics – that is, in the case of the Initiative, the numbers of activities reported by grantees, the laws adopted as per the GTER and the smoking prevalence as per the GATS – are often inflated. Indeed, given the predominance of ‘performance-based funding models’ in private–public global health ventures such as the Initiative, grantees ‘have strong incentives to over-report their performances’ (McCoy, Jensen *et al*., 2013, p. 2). Furthermore, ‘saved lives’ metrics tend to be too high because the estimates about the effectiveness of the public health interventions used – that is, in the case of the Initiative, estimates of the power of MPOWER policies – are too optimistic. This can be due to different reasons, including estimates being ‘derived from small-scale studies’ where the quality of interventions is ‘better than in real-world settings’; and a failure to factor in variations associated with different political, cultural and economic contexts or adjust for ‘different baseline mortality rates’, ‘patterns of disease’ and ‘relative risks of death’ (Levy, Ellis *et al*., 2013, p. 515; McCoy, Jensen *et al*., 2013, pp. 2–3). Finally, when there is a range of projects supporting similar public health interventions – as is the case in tobacco control, where the Initiative is one among a variety of other local, national and global anti-smoking efforts – there is a risk of double-counting and incorrectly attributing to oneself the lives saved thanks to the work of others.

## Epidemiology as the new bottom line

The values and practices developed by Bill Gates and Michael Bloomberg in their business ventures, their philanthropic work and, for Bloomberg, in public service offer insights into how audit and epidemiology, both alone and in combination, became central to the management of the Initiative. For both Gates and Bloomberg, information or data has been at the heart of their work at Microsoft Corporation and Bloomberg L.P., respectively. To start with, information and information technology was central to what their companies did. Writing in the 1990s, Gates (2008a, pp. 1, 4, 32) thought that the world was ‘moving into the information age’: a new, ‘exciting time’ when personalized computers and the internet – the ‘information highway’ – would make it possible to produce, store, analyse and transmit ever ‘more data’, thus revolutionizing every aspect of society, from business and government to health care, education and the home. He rightly viewed the work he and his colleagues were doing at Microsoft – developing programming languages, operating systems and other programmes – as a key driver of this revolution (Gates, 2008a, 2008b). Bloomberg, too, played an important role in the making of this new information age. Having started by selling financial data and analytical software through its own computer terminals, Bloomberg L.P. later expanded to become a world leader in the field of financial and business information (Bloomberg, 2001; Brash, 2011).

Moreover, echoing the cultures of audit deployed in the administration of commercial and corporate life in the twentieth century, Gates and Bloomberg also understood information as critical to good management and a successful business. Gates, in particular, was often viewed as ‘one of the high priests’ of this new culture of audit ‘built around the massive gathering and meticulous analysis of numerical data’, with managers at Microsoft expected to ‘crunch numbers for everything before they made decisions’ and ‘deliver quantifiable results for each dollar spent’ (Smith 2015, p. 148). For Gates (2008b, p. 4), ‘success’ in business ‘followed from positive leadership based on information and reason’. Good management, in other words, entailed the building of a complex ‘infrastructure’ to thoroughly and rigorously gather and analyse data about every aspect of one's business – from ‘information about product design and development’ and ‘marketing, sales and supplies’ to information ‘about payments and finance’ and ‘customer service’ (Gates, 2008b, p. 6). As Gates further explained:

Know your numbers is a basic business principle. You need to gather your business's data at every step of the way and in every interaction with your customers – and with your partners, too. Then you need to understand what the data means … If you do not understand what is happening in your business factually, and you are making decisions based on soft data or emotion, you will eventually pay a big price. Numbers give you the factual basis for the directions in which you take your products … They help you identify your highest priorities. (Gates, 2008b, pp. 62–63)

Bloomberg had very similar values and beliefs, repeatedly stressing that ‘following the data’ was the motto at the heart of his company and famously stating that: ‘In God we trust; everyone else bring data’ (Bloomberg Philanthropies, 2013, p. 3).

Unsurprisingly perhaps, Gates and Bloomberg brought their belief in data and audit that had been so central to their businesses over into their philanthropic work. Discussing the similarities and differences between his work at Microsoft and running a foundation, Gates declared that many of the lessons he had learned in business, and especially his concern with data and measurement, would apply to philanthropy:

The common sense of the business world, with its urgency and focus, has strong application in the philanthropic world. I am sure I will make mistakes in over-applying some elements from my previous experience and will need to adjust … However, I am equally confident that our maniacal focus on … measuring results will make a difference. (Gates, 2009, p. 2)

You can achieve amazing progress [in your philanthropic endeavours] if you set a clear goal and find a measure that will drive progress towards that goal … Given a goal, you decide on what key variable you need to change to achieve it – the same way a business picks objectives for inside the company … – and develop a plan for change and a way of measuring the change. You use the measurement as feedback to make adjustments. (Gates, 2013, pp. 2–3)

Bloomberg was, if anything, even more confident about applying the insights he picked up in his business and, specifically, his belief in data and evaluation to his philanthropic work:

Data is the driving force behind my company's value to the financial services industry … Bloomberg Philanthropies is no different. It harnesses the power of data to assess opportunities, understand impact, evaluate results and improve performance. (Bloomberg Philanthropies, 2013, p. 3)

Of course, Gates and Bloomberg were not the only ones applying their belief in the power of data and audit acquired in the business world to their philanthropic work. Rather, as Matthew Bishop and Michael Green (2008, pp. 1–2) have argued, they were illustrative of a wider trend whereby ‘today's new philanthropists are trying to apply the secrets behind their money-making success to their giving’ and which they termed ‘philanthro-capitalism’. And, what is important for us is that it is as part of this wider trend that a culture of audit articulated around reports, evaluations and management experts made its way into the Bloomberg Initiative and other philanthropic efforts. To borrow from McCoy and McGoey (2011, p. 146–147), ‘the application of competitive business skills, acumen, and tactics to philanthropy and charitable activities’ has led, among other things, to a ‘greater insistence on measurable results and more direct management of grantees by their funders’ (see also Mahajan, in press; Rushton & Williams, 2011; Smith, 2015).

It is also because of Gates and Bloomberg's belief in data and measurement that epidemiological expertise and practices became so central to the Bloomberg Initiative. But here the precise pathways differ for the two philanthropists. For Gates, epidemiology was the response to his search for the ultimate yardstick of success or failure in his philanthropic work on global health. Gates quickly realized that while he could transfer the techniques of audit like reports, indicators and evaluations from his business to his foundation, he could not do the same with the notion of profit. Profit in business was the bottom-line, the definitive measure of whether what you did worked or not. The fact that it did not apply to philanthropic work was a problem for Gates in so far as he was left without any criteria to judge whether his efforts were making a difference or not. As he argued:

Unlike business, where profit is the bottom line, foundations … pick their own goals [and make that their bottom line]. (Gates, 2013, p. 2)

[A] way that running a foundation is not like running a business is that you don't have customers who beat you up when you get things wrong or competitors who work to take those customers away from you. You don't have a stock price that goes up and down to tell you how you are doing. This lack of natural feedback loop means that we as a foundation have to be even more careful in picking our goals and being honest with ourselves when we are not achieving them. We work hard to get … feedback. (Gates, 2009, p. 14)

This explains why the Gates Foundation has spent a lot of time and money in identifying clear, measurable alternative ‘bottom lines’ to assess its philanthropic investments. In the field of education, this has led it to get states in the United States to ‘agree to common rules on how to measure graduation rates and on what standards must be achieved to graduate’ (Bishop & Green, 2008, p. 60). In the field of global health, it has led the Foundation to focus on the numbers of lives saved and fund Christopher Murray and his team at the Institute of Health Metrics and Evaluation to calculate the global burden of disease and disability at regular intervals (Smith, 2015). As Gates explains:

The metric of success is lives saved … Which is slightly different than units sold [or] profits achieved. But it's all very measurable [through epidemiology], and you can set ambitious goals and see how you do. (Gates cited in Smith, 2015, p. 149)

For Bloomberg, in contrast, his discovery and interest in epidemiology developed during his time as Mayor of New York City. As Julian Brash (2011) explains, Bloomberg brought his faith in data and evaluation, which he had acquired in his business, with him when he took over at City Hall. Inspired by a rhetoric of transparency and efficiency derived from New Public Management and seeking to be a ‘CEO Mayor’ as he had promised during his electoral campaign, he set up multiple systems to monitor and measure the city's public services. Some of these systems were internal and for the benefit of the administration, like the Citywide Accountability Programme, a scheme providing managers information to assess and improve their employees’ performances. Others were external and for the benefit of citizens. There was, for example, the 311 phone helpline where citizens could complain and report problems and which, together with citywide customer satisfaction surveys, generated data about citizens’ level of contentment about municipal services. And there was the Mayor's Public Report Card, an annual report with goals and targets as well as indicators and result-oriented statistics demonstrating success or failure. It is as part of these efforts at transparency and efficiency at City Hall that Bloomberg started engaging with epidemiological expertise and practices. Indeed, his Health Commissioner, Tom Frieden, and his team at the Department of Health collected many health statistics – including on smoking prevalence and attitudes to tobacco use – which Bloomberg and his advisors consulted and integrated in their annual Public Report Cards. From there, epidemiological expertise and practices went on to influence the Bloomberg Initiative thanks to the central role played by Frieden and others in the design of the Initiative. As one expert working for the latter explained, there was a strong alliance between Bloomberg and epidemiologists based on their shared belief in data:

Bloomberg is 100% about data. His entire business is built on data. At City Hall he implemented all sorts of data-driven initiatives. This is a person very interested in data. Similarly, I am a trained epidemiologist and my focus is also on data … We both just want to know what impact we are having. That is why we fund measurement strategies like the GATS.

## Conclusion

Contrary to what is often argued in the more critically minded literature on global health, I suggested that philanthropists’ growing influence in the organization and management of world health has not led to the disappearance of all forms of accountability. Specifically, and focusing on the Bloomberg Initiative spearheaded by the Bloomberg and Gates foundations, I have argued that the increasing involvement of philanthropists in global health has led to the development of a new form of accountability – epidemiological accountability – which combines the expertise and practices of audit with those of modern epidemiology. In these concluding remarks, I want to draw some of the broader implications that my argument might have for those of us in the social sciences and humanities who work on and write about the languages and cultures of accountability that permeate the fields of global health, international development and beyond.

When faced with a new form of accountability like epidemiological accountability, one question that some might be tempted to ask is whether this new form of accountability works at all. This, I believe, would be asking the wrong question. Indeed, such a question fails to unpack the key ideas and practices – the desire to save lives; the drive for effectiveness; the trust in numbers; the monitoring systems and evaluation procedures – around which epidemiological accountability is articulated. Put differently, to ask such a question would mean evaluating whether this new form of accountability really makes saving lives and the management of world health more effective, thus very much operating from within the epidemiological accountability episteme and reproducing rather than interrogating its core assumptions. A more fruitful approach, I suggest, would be to ask what this new form of accountability produces. This question shifts our analytical gaze from epidemiological accountability's possible shortcomings to its performative power and the new forms of knowledge and subjectivity it generates (Desrosières, 2015; Power, 1997). As I showed in the paper, epidemiological accountability helps create many such new forms. A good example is the ‘saved lives’ metrics which the Bloomberg and Gates foundations regularly use to assess which parts of the Initiative are working and which ones are not. This new type of metrics is based on complex computer simulation models which combine into one numerical estimate all the different data captured by the Initiative's audit and surveillance systems – Bloomberg and Gates's investments, the grantees’ self-reported activities, national tobacco control laws, smoking prevalence rates and the like. Another great example is the figure of the professional advocate who is not just passionate about tobacco control and global health but also fully trained in project management – from filling in time sheets and writing up reports to managing a budget and running an organization. This new type of subject emerges out of, to quote from Gilles Deleuze's (1992, p. 7) *Postscript on the societies of control*, the ‘continuous forms of control’ and the ‘perpetual training’ logic associated with the Initiative – the constant self-recording and reporting; the regular inspections during which accountants and management experts offer advice and counsel on how to organize and work; and the training courses in project management and accounting (see also Jensen & Winthereik, 2013).

Another aspect of epidemiological accountability that I would like to emphasize is the way it has transformed audit from a profit-making tool of late-twentieth-century corporate capitalism into a biopolitical instrument of modern philanthropy. This transformation is particularly evident when one compares the culture of corporate accountability that Bill Gates put in place at Microsoft with the practices of epidemiological accountability permeating the Initiative. As I suggested in this paper, both share similar audit techniques and expertise. Indeed, like Bloomberg and other philanthropists, Bill Gates made very clear that he wanted to bring his business acumen and know-how – including and especially his belief in data and audit practices – into his philanthropic work. But, as he made clear himself, there is an important difference between the audit practices used at Microsoft and those at work within the Initiative: at Microsoft, audit practices are a tool for making profit; in the Initiative, these same practices are an instrument to save lives – an audit of saving lives, if you will. This shift from profit-making to saving lives is made possible by the combination of audit practices with epidemiological techniques that is so characteristic of epidemiological accountability. An excellent illustration is the computer simulation models mentioned in the previous paragraph, which associate the grantees’ activities captured through audit and the numbers of lives saved measured through epidemiological proxies like smoking prevalence. It is worth noting that the way epidemiological accountability de-couples audit practices from profit and re-couples them with life directly contradicts the claim made by anthropologist Susan Erikson (2016) that the private turn in global health has transformed health metrics into tools for profit-making. It is also worth emphasizing that epidemiological accountability differs from the audit practices permeating international development described by Rottenburg (2000) and others (e.g. Jensen & Winthereik, 2013; Merry, 2011). Indeed, as some commentators have pointed out (Edwards & Hulme, 1996, p. 968; see also Fowler, 1996), the audit practices deployed in the field of development often have ‘no obvious bottom line’ – a consequence of having been de-coupled from the profit motive but not re-coupled with other measures of success, in contrast to epidemiological accountability.

To finish, I would like to reflect on the figure of the accountee in epidemiological accountability and, in this way, return to the body of literature critiquing philanthropies’ lack of accountability explored at the start of the paper.^4^ All forms of accountability presume a particular kind of accountee, that is an imagined public or group of persons to whom one has to give account and who will appraise this account (Bovens, 2006; Hesselmann, 2011; Power, 1997). To capture the specific nature of the imagined public of epidemiological accountability in the Initiative, I want to compare it with the figures of the accountee informing two other forms of accountability: corporate accountability at Microsoft when Bill Gates was CEO; and democratic accountability in New York City when Michael Bloomberg was Mayor. At Microsoft, the accountees are the shareholders or investors: they read the company's annual reports to assess whether their investments are profitable and, if not, they divest. In Bloomberg's New York, the accountees are the citizens: they examine the Public Report Cards issued by the Mayor and, if they are dissatisfied with the way he runs the city, they vote him out. In contrast to Microsoft's investors and New York's citizens, the imagined publics of the Bloomberg Initiative are global health experts. These experts can examine the epidemiological and audit data collected for the Initiative, much of which is made public in documents like the Bloomberg and Gates foundations’ annual reports, the WHO's Reports on the Global Tobacco Epidemic and the CDC's Global Adult Tobacco Surveys. And, if they have concerns, they can voice these in reasoned critiques published in academic journals or presented at scientific conferences. An excellent and, for some, perhaps unexpected example is the very commentators who have critiqued philanthropies’ lack of accountability and whose work we examined at the beginning. Indeed, these commentators – like *The Lancet* editor Richard Horton and Oxford professor David Stuckler – are prominent global health experts who have developed and published in leading journals sophisticated and powerful critiques of philanthropies like the Gates and Bloomberg foundations. Contrary to what they seem to think, it is them and their reasoned critiques – rather than an elusive world citizenry and a utopian global democratic system – that can and already have forced philanthropists to listen and change the way they work.

## Notes

1 Commentators who bemoan the lack of transparency and accountability displayed by philanthropies working in global health do not usually employ the term ‘social accountability’, preferring instead the expressions ‘democratic’ or ‘public accountability’ (e.g. McCoy & McGoey, 2011, p. 157; Rushton & Williams, 2011, p. 18). However, there is no doubt that what these commentators have in mind when they speak about ‘democratic’ or ‘public accountability’ is the same thing as what the World Bank and many development experts like to call ‘social accountability’ (Malena *et al*., 2004).

2 This research project draws on three different types of data: (i) papers, books, reports, pamphlets and other documents authored by individuals and organizations associated with the Bloomberg Initiative in one way or another; (ii) semi-structured interviews with over 100 experts, activists, managers and other individuals working for or otherwise involved with the Initiative; and (iii) ethnographic observations recorded at workshops, meetings and other events related to the Initiative and attended by the author. This corpus of texts, interviews and notes was examined to identify and make sense of the cultures and practices of accountability that inform and shape the organization and management of the Initiative (see Hine, 2007; Latour, 1993; Prainsack & Wahlberg, 2013).

3 In late 2014, the Gates Foundation sold its shares in Coca-Cola and McDonald's, keeping, however, its holdings in other big players within the alcohol and food industry (Cohen, 2015).

4 Accountee is a term that I have coined to describe the group of persons to whom one has to give account and who will pass judgement on this account. It is synonymous with ‘principal’, ‘accountability taker’ and ‘accountability forum’, which are the terms generally used in the specialized literature on accountability but which I found uninformative, confusing and/or somewhat inelegant (Bovens, 2006; Hesselmann, 2011; Power, 1997). I coined the term by amalgamating ‘account’ with the suffix ‘–ee’. According to the Oxford English Dictionary, the suffix ‘–ee’ comes from the Anglo-Norman and Old French ‘–é’ and has been ‘freely added to English verb stems to form nouns denoting the passive party’ while, at the same time and in contrast, the prefix ‘–or’ has been added to the same verb stems to denote the active agent, as in grantee/grantor, trustee/trustor and tutee/tutor. By the same logic, the person who gives account could be termed the accountor.
